# Surgical Repair vs Splenectomy in Patients With Severe Traumatic Spleen Injuries

**DOI:** 10.1001/jamanetworkopen.2024.25300

**Published:** 2024-08-02

**Authors:** Dominik A. Jakob, Martin Müller, Apostolos Kolitsas, Aristomenis K. Exadaktylos, Demetrios Demetriades

**Affiliations:** 1 Division of Trauma and Surgical Critical Care, Department of Surgery, Los Angeles General Medical Center, University of Southern California, Los Angeles; 2 Department of Emergency Medicine, Inselspital Bern University Hospital, University of Bern, Bern, Switzerland; 3 Department of Visceral Surgery, Lindenhofspital, Bern, Switzerland

## Abstract

**Importance:**

The spleen is often removed in laparotomy after traumatic abdominal injury, with little effort made to preserve the spleen.

**Objective:**

To explore the association of surgical management (splenic repair vs splenectomy) with outcomes in patients with traumatic splenic injuries undergoing laparotomy and to determine whether splenic repair is associated with lower mortality compared with splenectomy.

**Design, Setting, and Participants:**

This is a trauma registry–based cohort study using the American College of Surgeons Trauma Quality Improvement Program database from January 2013 to December 2019. Participants included adult patients with severe splenic injuries (Abbreviated Injury Scale [AIS] grades 3-5) undergoing laparotomy after traumatic injury within 6 hours of admission. Data analysis was performed from April to August 2023.

**Exposures:**

Splenic repair vs splenectomy in patients with severe traumatic splenic injury.

**Main Outcomes and Measures:**

The primary outcome was in-hospital mortality. Outcomes were compared using different statistical approaches, including 1:1 exact matching with consecutive conditional logistic regression analysis as the primary analysis and multivariable logistic regression, propensity score matching, and inverse-probability weighting as sensitivity analyses.

**Results:**

A total of 11 247 patients (median [IQR] age, 35 [24-52] years; 8179 men [72.7%]) with a severe traumatic splenic injury undergoing laparotomy were identified. Of these, 10 820 patients (96.2%) underwent splenectomy, and 427 (3.8%) underwent splenic repair. Among patients who underwent an initial splenic salvage procedure, 23 (5.3%) required a splenectomy during the subsequent hospital stay; 400 patients with splenic preservation were matched with 400 patients who underwent splenectomy (matched for age, sex, hypotension, trauma mechanism, AIS spleen grade, and AIS groups [0-2, 3, and 4-5] for head, face, neck, thorax, spine, and lower and upper extremity). Mortality was significantly lower in the splenic repair group vs the splenectomy group (26 patients [6.5%] vs 51 patients [12.8%]). The association of splenic repair with lower mortality was subsequently verified by conditional regression analysis (adjusted odds ratio, 0.4; 95% CI, 0.2-0.9; *P* = .03). Multivariable logistic regression, propensity score matching, and inverse-probability weighting confirmed this association.

**Conclusions and Relevance:**

In this retrospective cohort study, splenic repair was independently associated with lower mortality compared with splenectomy during laparotomy after traumatic splenic injury. These findings suggest that efforts to preserve the spleen might be indicated in selected cases of severe splenic injuries.

## Key Points

**Question:**

Is splenic repair associated with better outcomes compared with splenectomy in patients with traumatic splenic injury undergoing laparotomy?

**Findings:**

In this cohort study of 11 247 patients, splenic repair was independently associated with lower mortality compared with splenectomy during laparotomy.

**Meaning:**

These findings suggest that efforts to preserve the spleen should be made in selected cases of patients with traumatic splenic injuries undergoing laparotomy.

## Teaser

This cohort study examines the association of surgical management with splenic repair vs splenectomy with outcomes in patients with traumatic splenic injuries undergoing laparotomy.

## Introduction

The spleen is one of the most frequently injured organs in cases of abdominal trauma.^1^ Although selective nonoperative management of splenic injury has become the standard of care for stable patients with trauma, surgical intervention is still frequently required for associated intraabdominal injuries or severe abdominal hemorrhage.^1,2,3,4^ According to recent studies and data from the American College of Surgeons National Trauma Data Bank,^5,6^ up to 20% of patients admitted with splenic injury still undergo urgent laparotomy.

Currently, there has been a practice to manage splenic injuries, even severe ones, nonoperatively by using supportive measure such as transfusions and angioembolization. However, in patients undergoing laparotomy, there has been a low threshold for splenectomy, even for relatively minor splenic injuries, which is a change of practice over the last few decades. Ko et al^7^ recently compared a contemporary cohort including patients from 2014 to 2018 with a historical cohort including patients from 1980 to 1989. Splenorrhaphy was performed for 43.4% of patients in the historical cohort and for only 1.4% of patients in the contemporary cohort, whereas the success rate of splenorrhapy has not changed over time (98.7% vs 100%). Another recent National Trauma Data Bank study^5^ confirmed the low rate (1.7% in 2015) of splenic repairs for splenic injuries. However, there are very limited data available on how surgical strategy affects outcomes.

The aim of the present study was to evaluate the association of different surgical strategies (splenic repair vs splenectomy) with outcomes in patients with traumatic injury undergoing laparotomy. We hypothesized that splenic repair is associated with better outcomes compared with splenectomy. Our hypothesis is based on the following potential factors: first, the absence of the spleen as an important organ in the immune system can increase patients’ susceptibility to infections and may increase postoperative complications in general^8^; and second, splenectomy may increase the risk of venous thromboembolism, because the spleen plays an important role in regulating blood flow and removing aged or damaged blood cells.^9^

## Methods

This cohort study was approved by the institutional review board of the University of Southern California. This study follows the Strengthening the Reporting of Observational Studies in Epidemiology (STROBE) reporting guidelines. Informed consent was not obtained because the data are deidentified, in accordance with 45 CFR §46.

### Study Design and Setting

This is a trauma registry–based cohort study using the American College of Surgeons Trauma Quality Improvement Program (TQIP) database from January 2013 to December 2019. The TQIP database is maintained by the American College of Surgeons Committee on Trauma and aggregates patient data from more than 760 trauma centers across the US.^10^

### Participants: Eligibility Criteria

The database was queried to identify all adult patients (aged ≥16 years old) who sustained severe splenic injury, defined as an Abbreviated Injury Scale (AIS) score of 3 to 5 (range, 1 [minor] to 6 [maximal]). Exclusion criteria included hospital transfers and missing information on splenic procedure timing, age, sex, systolic blood pressure, heart rate, and Glasgow Coma Scale (GCS) score (range, 3 [worst] to 15 [best]).

Only patients who underwent laparotomy with splenic repair or splenectomy within 6 hours of admission were considered for final analysis. Patients who underwent splenectomy after initial splenic repair were considered as having a failed splenic repair.

### Variables

Patient variables were extracted from the TQIP database (refer to the eAppendix and eTable 1 in Supplement 1 for detailed definitions). Demographic characteristics included age, sex, race (Asian, Black, White, and any other race not otherwise specified), height (centimeters), and weight (kilograms). In the TQIP database, race is self-reported by patients or identified by a family member; data on race are included here because race may be a potential confounder for our primary end point and can, therefore, be considered in the analysis. Comorbidities included steroid use, current smoker, diabetes, hypertension, cerebrovascular accident, respiratory disease, congestive heart failure, myocardial infarction, liver cirrhosis, chronic kidney disease, peripheral arterial disease, disseminated cancer and/or receipt of chemotherapy, dementia, substance abuse disorder, bleeding disorder, and anticoagulation therapy. Admission data included heart rate, systolic blood pressure, GCS score, and mechanism of injury (blunt vs penetrating), including treatment at a level I trauma center. Injury data included anatomic location and severity of injury using the AIS. Details on surgical splenic procedures, including procedure timing, were also included.

### Outcomes

The primary outcome was in-hospital mortality. Secondary outcomes were complications (thromboembolic events, stroke, myocardial infarction, acute kidney injury, acute respiratory distress syndrome, sepsis, and unplanned return to the operating room), intensive care unit (ICU) admission, length of hospital stay, and length of ICU stay.

### Bias and Study Size

As quality control, the final dataset and splenic procedures were obtained independently by 2 researchers (D.A.J. and M.M.) using SPSS statistical software version 26.0 (IBM) and Stata/MP statistical software version 18.0 (StataCorp), respectively. All eligible patients were included. No a priori sample size calculation was performed.

### Univariable and Multivariable Conditional Regression Analysis After 1:1 Exact Matching

A 1:1 exact matching of patients undergoing splenic repair vs splenectomy within 6 hours of admission was performed on the basis of the following important clinical criteria: age groups (16-45, >45 to 65, >65 to 75, and >75 years), sex, hypotension (blood pressure <90 mm Hg) at admission, trauma mechanism (blunt vs penetrating), AIS spleen grades (3, 4, and 5) and AIS groups (0-2, 3, and 4-5) for head, face, neck, thorax, spine, and lower and upper extremity. Patients for whom splenic repair failed were still included in the splenic repair group for analysis (intention-to-treat). Primary and secondary outcomes between the 2 groups were subsequently compared with univariable and multivariable conditional regression analysis.

### Statistical Analysis

Data analysis was performed from April to August 2023. In the total cohort, categorical variables between patients who underwent splenic repair and splenectomy were compared using χ^2^ test. Wilcoxon rank sum test was used to compare the distributions for continuous variables between the 2 groups. Results are reported as numbers and percentages for categorical variables or medians and IQRs for continuous variables. Results are reported as adjusted odds ratios (aORs) and 95% CIs.

In the matched cohort, the association of the surgical strategy was further analyzed with conditional multivariable logistic regression analysis. For univariable comparison in the matched cohort, Wilcoxon signed rank tests for continuous and McNemar tests (exact *P* values) for binary outcomes were used. All variables with *P *< .20 on univariable analysis associated with the exposure (splenic repair vs splenectomy) were included as covariables for the conditional regression analysis.

For sensitivity analysis, multivariable logistic regression analysis, propensity score matching (PSM), and inverse probability weighting (IPW) were also used to assess the effect of splenic repair compared with splenectomy on mortality (primary outcome). All variables with *P *< .20 on univariable analysis in the total cohort associated with the exposure were included into different statistical models: (1) multivariable logistic regression, (2) PSM, and (3) IPW using the total cohort. The effect size for PSM with respect to IPW was the average treatment effect (ATE) (with the treatment defined as splenectomy) with 95% CIs. Balancing was checked with the tebalance summarize command in Stata using standardized differences. The ATE for mortality is the proportional change in mortality comparing splenectomy with splenic repair. The main and sensitivity analyses were applied to all primary and secondary outcomes.

Variables with 2-sided *P* < .05 were considered significant. Stata/MP statistical software version 18.0 (StataCorp) was used for statistical analysis.

## Results

A total of 11 247 patients (median [IQR] age, 35 [24-52] years; 8179 men [72.7%]) with a severe splenic injury (AIS grades 3-5) undergoing trauma laparotomy within 6 hours of admission were identified. Of these, 10 820 patients (96.2%) underwent splenectomy and 427 patients (3.8%) had splenic repair within 6 hours of admission (Figure).

**Figure.**
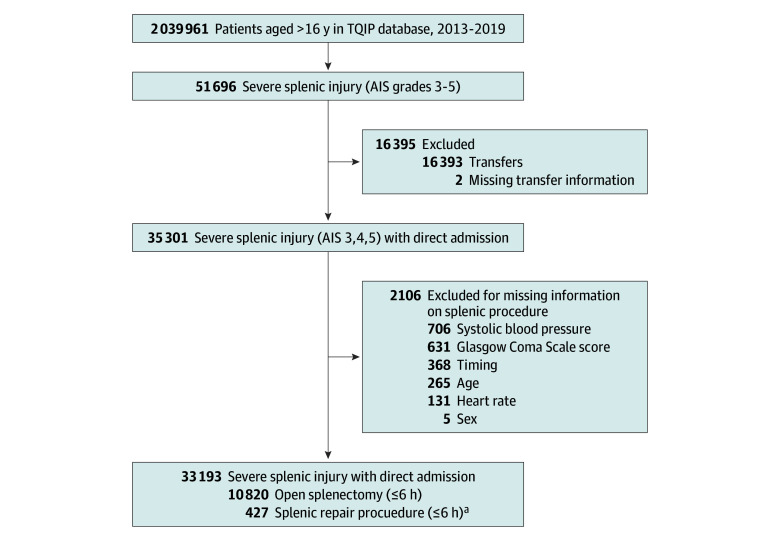
Patient Enrollment Flowchart AIS indicates Abbreviated Injury Scale; TQIP, Trauma Quality and Improvement Program. ^a^Of these, 23 patients (5.4%) had a splenectomy in the follow-up.

### Cohort Characteristics in Patients With Splenic Repair vs Splenectomy

eTable 2 in Supplement 1 shows the prematched analysis of patients with splenic repair and those who underwent initial splenectomy. Among patients who underwent an initial splenic salvage procedure, 23 (5.3%) underwent a splenectomy during the subsequent hospital stay (failed splenic repair group). Four patients (17.4%) in the failed repair group died compared with 1795 patients (16.6%) in the initial splenectomy group.

### Univariable and Multivariable Conditional Regression Analysis After 1:1 Exact Matching

Four hundred of 427 patients (93.7%) with splenic repair were 1:1 matched with 400 of 10 820 control patients (3.7%) who underwent splenectomy within 6 hours of admission. In the matched cohort, 17 patients (4.3%) underwent a splenectomy after initial splenic repair (failed splenic repair). Two of 17 patients (11.8%) for whom splenic repair failed died compared with 51 of 400 patients (12.8%) in the initial splenectomy group. Epidemiologic and clinical characteristics (Table 1), as well as trauma type and injury severity (eTable 3 in Supplement 1), were similar between the 2 groups after matching on the basis of the aforementioned criteria. Mortality was 6.5% (26 patients) among patients who had splenic repair compared with 12.8% (51 patients) among patients who underwent splenectomy. Secondary outcomes were similar between the 2 groups after 1:1 matching (Table 2). The subsequent conditional regression analysis (including the following covariables: race, current smoking, diabetes, AIS abdomen excluding the spleen, and Injury Severity Score [range, 3-75, with higher scores indicating worse severity]) showed a negative association of splenic repair with mortality (aOR, 0.4; 95% CI, 0.2-0.9; *P* = .03) (Table 3).

**Table 1.** Patients’ Epidemiological and Clinical Characteristics After 1:1 Exact Matching^a^

Characteristic	Patients, No. (%)		*P* value^b^
	Splenectomy (n = 400)	Splenic repair (n = 400)	
Demographics			
Age, median (IQR), y	31 (23-44)	30 (23-44)	.40
Age groups, y			
16-45	312 (78.0)	312 (78.0)	>.99
>45-65	67 (16.8)	67 (16.8)	>.99
>65-75	12 (3.0)	12 (3.0)	>.99
>75	9 (2.2)	9 (2.2)	>.99
Sex			
Male	304 (76.0)	304 (76.0)	>.99
Female	96 (24.0)	96 (24.0)	>.99
Body mass index >30^c^	86 (21.5)	90 (22.5)	.73
Race			
Asian	7 (1.8)	7 (1.8)	>.99
Black	114 (28.5)	93 (23.2)	.07
White	230 (57.5)	226 (56.5)	.82
Other^d^	49 (12.2)	74 (18.5)	.03
Level I trauma center treatment	220 (55.2)	212 (53.1)	.21
Clinical characteristics at admission			
Hypotension (systolic blood pressure <90 mm Hg)	56 (14.0)	56 (14.0)	>.99
Tachycardia (heart rate >120 beats/min)	94 (23.5)	85 (21.2)	.47
Glasgow Coma Scale score, median (IQR)	15 (14-15)	15 (14-15)	.89
Comorbidities			
Any comorbidities	192 (48.0)	180 (45.0)	.43
Steroid use	3 (0.8)	1 (0.2)	.63
Current smoker	109 (27.3)	91 (22.8)	.14
Diabetes	26 (6.5)	15 (3.8)	.11
Hypertension	59 (14.8)	49 (12.2)	.33
Cerebrovascular accident	3 (0.8)	2 (0.5)	>.99
Respiratory disease	16 (4.0)	13 (3.2)	.71
Congestive heart failure	6 (1.5)	2 (0.5)	.22
Myocardial infarction (past)	3 (0.8)	1 (0.2)	.63
Liver cirrhosis	5 (1.2)	1 (0.2)	.22
Chronic kidney disease	0	2 (0.5)	.50
Peripheral arterial disease	0	2 (0.5)	.50
Disseminated cancer and/or receiving chemotherapy	0	1 (0.2)	>.99
Dementia	0	0	>.99
Substance abuse disorder	66 (16.5)	68 (17.0)	.93
Bleeding disorder	5 (1.2)	6 (1.5)	.76
Anticoagulant therapy	6 (1.5)	3 (0.8)	.32

^a^ Of 427 patients with splenic repair, 400 (93.7%) were 1:1 matched with control patients who had splenectomy within 6 hours of admission. The controls were matched on age groups (16-45, >45 to 65, >65 to 75, and >75 years), sex, hypotension (systolic blood pressure <90 mm Hg) at admission, penetrating trauma mechanism, and Abbreviated Injury Scale grades (3-5) for spleen, and Abbreviated Injury Scale groups (0-2, 3, and 4-5) for head, face, neck, thorax, spine, and lower and upper extremity.

^b^ *P* values were calculated using Wilcoxon signed rank test and McNemar test (exact *P* values).

^c^ Body mass index is calculated as weight in kilograms divided by height in meters squared.

^d^ Any races not classified as White, Black, or Asian were classified as other race.

**Table 2.** Complications and Outcomes After 1:1 Exact Matching^a^

Complications and outcomes	Patients, No. (%)		*P* value^b^
	Splenectomy (n = 400)	Splenic repair (n = 400)	
Complications			
Any complications	68 (17.0)	65 (16.2)	.84
Thromboembolic event	13 (3.2)	16 (4.0)	.69
Deep vein thrombosis	8 (2.0)	9 (2.2)	>.99
Pulmonary embolism	5 (1.2)	8 (2.0)	.55
Stroke	0 (0.0)	3 (0.8)	.25
Myocardial infarction	1 (0.2)	2 (0.5)	>.99
Acute kidney injury	19 (4.8)	13 (3.2)	.35
Acute respiratory distress syndrome	8 (2.0)	8 (2.0)	>.99
Pneumonia	30 (7.5)	28 (7.0)	.88
Severe sepsis	14 (3.5)	7 (1.8)	.19
Unplanned return to operating room	24 (6.0)	19 (4.8)	.52
Outcomes			
Length of hospital stay, d	10 (6-17)	9 (6-18)	.99
ICU treatment needed	334 (83.5)	317 (79.2)	.12
ICU treatment duration, d	4 (1-9)	3 (1-8)	.65
In-hospital mortality	51 (12.8)	26 (6.5)	.002

Abbreviation: ICU, intensive care unit.

^a^ Of 427 patients with splenic repair, 400 (93.7%) were 1:1 matched with control patients who had splenectomy within 6 hours of admission. The controls were matched on age groups (16-45, >45 to 65, >65 to 75, and >75 years), sex, hypotension (systolic blood pressure <90 mm Hg) at admission, penetrating trauma mechanism, Abbreviated Injury Scale grades (3-5) for spleen, and Abbreviated Injury Scale groups (0-2, 3, and 4-5) for head, face, neck, thorax, spine, and lower and upper extremity.

^b^ *P* values were calculated using Wilcoxon signed rank test and McNemar test (exact *P* values).

**Table 3.** Effect Sizes of Splenic Repair (n = 427) vs Splenectomy (n = 10 820) on Mortality in Patients With Severe Splenic Trauma Using Different Statistical Approaches

Statistical approach	Effect size (95% CI)	*P* value
1:1 Exact matching and consecutive multivariable conditional logistic regression^a^	aOR**,** 0.4 (0.2 to 0.9)	.03
Multivariable logistic regression^b^	aOR, 0.6 (0.4 to 0.9)	.01
Propensity score matching^b^	ATE, −0.05 (−0.09 to −0.02)	.003
Inverse-probability weighting^b^	ATE, −0.05 (−0.09 to −0.01)	.009

Abbreviations: aOR, adjusted odds ratio; ATE, average treatment effect.

^a^ Covariables included race as categorical parameter, smoking, diabetes, Abbreviated Injury Scale group (0-2, 3, and 4-5) for abdomen excluding spleen, and Injury Severity Score.

^b^ Covariables included age group, race, hypotension (systolic blood pressure <90 mm Hg), tachycardia (heart rate >120 beats/min), Glasgow Coma Scale score, liver cirrhosis, chronic kidney disease, penetrating trauma, Injury Severity Score, and Abbreviated Injury Scale scores for head, face, neck, spleen, abdomen without spleen, spine, and lower and upper extremity.

Table 4 summarizes the effect sizes comparing splenic repair (exposure) with splenectomy (baseline) with regard to the primary and secondary outcomes and complications. No consistent significant associations were found apart from mortality. However, the conditional regression after 1:1 matching including the aforementioned covariables revealed a significantly lower ICU admission rate among patients who underwent splenic repair vs those who underwent splenectomy (aOR, 0.6; 95% CI, 0.4-1.0; *P* = .04). Patients who underwent splenic repair also had a lower, but not statistically significant, aOR for severe sepsis (aOR, 0.1; 95% CI, 0.0-1.1; *P* = .06) compared with patients who underwent splenectomy.

**Table 4.** Association of Splenic Repair vs Splenectomy With Complications and Primary and Secondary Outcomes in Severe Splenic Trauma Using Different Statistical Approaches^a^

Complications and outcomes	1:1 Exact matching and consecutive multivariable conditional logistic regression		Multivariable logistic regression		Propensity score matching		Inverse-probability weighting	
	aOR (95% CI)	*P* value	aOR (95% CI)	*P* value	ATE (95% CI)	*P* value	ATE (95% CI)	*P* value
Complications								
Any complications	1.1 (0.7 to 1.8)	.68	0.8 (0.6 to 1.1)	.16	0.00 (−0.05 to 0.05)	.99	0.00 (−0.06 to 0.07)	.99
Thromboembolic event	1.3 (0.5 to 3.9)	.61	0.7 (0.5 to 1.2)	.19	−0.00 (−0.03 to 0.03)	.96	0.00 (−0.04 to 0.04)	.98
Deep vein thrombosis	1.3 (0.3 to 5.3)	.73	0.6 (0.3 to 1.1)	.08	−0.00 (−0.03 to 0.03)	.81	0.00 (−0.04 to 0.04)	1.00
Pulmonary embolism	NA	NA	0.9 (0.5 to 1.8)	.88	−0.00 (−0.02 to 0.02)	.91	−0.00 (−0.02 to 0.02)	.98
Stroke	NA	NA	0.8 (0.3 to 2.8)	.78	−0.00 (−0.01 to 0.00)	.30	−0.00 (−0.01 to 0.01)	.42
Myocardial infarction	NA	NA	0.9 (0.2 to 3.9)	.91	−0.00 (−0.01 to 0.01)	.86	0.00 (−0.01 to 0.01)	.87
Acute kidney injury	0.9 (0.3 to 3.1)	.85	0.9 (0.5 to 1.5)	.66	−0.00 (−0.03 to 0.02)	.83	−0.01 (−0.03 to 0.01)	.32
Acute respiratory distress syndrome	NA	NA	0.7 (0.4 to 1.4)	.35	−0.02 (−0.03 to 0.00)	.07	−0.02 (−0.03 to −0.00)	.03
Pneumonia	1.0 (0.5 to 2.2)	.99	1.2 (0.8 to 1.8)	.36	0.01 (−0.03 to 0.05)	.55	0.02 (−0.03 to 0.06)	.45
Severe sepsis	0.1 (0.0 to 1.1)	.06	0.8 (0.4 to 1.6)	.53	−0.00 (−0.02 to 0.02)	.90	−0.00 (−0.02 to 0.02)	.91
Unplanned return to operation room	1.5 (0.5 to 4.5)	.45	0.8 (0.5 to 1.2)	.28	0.02 (−0.01 to 0.05)	.30	0.01 (−0.03 to 0.06)	.57
Primary and secondary outcomes								
In-hospital mortality	0.4 (0.2 to 0.9)	.03	0.6 (0.4 to 0.9)	.01	−0.05 (−0.09 to −0.02)	.003	−0.05 (−0.09 to −0.01)	.009
Any complications including death	0.7 (0.5 to 1.2)	.23	0.7 (0.5 to 0.9)	.005	−0.06 (−0.11 to −0.00)	.04	−0.05 (−0.12 to 0.01)	.11
Length of hospital stay, d	0.5 (−1.5 to 2.5)	.61	−0.8 (−2.3 to 0.7)	.30	1.06 (−0.85 to 2.98)	.28	0.62 (−1.45 to 2.69)	.56
ICU treatment needed	0.6 (0.4 to 1.0)	.04	0.8 (0.6 to 1.1)	.17	0.01 (−0.01 to 0.04)	.33	0.02 (−0.02 to 0.06)	.39
ICU treatment duration, d	0.5 (−0.9 to 1.8)	.49	−0.0 (−1.0 to 1.0)	.99	0.54 (−0.74 to 1.83)	.41	0.34 (−0.98 to 1.66)	.61

Abbreviations: aOR, adjusted odds ratio; ATE, average treatment effect; ICU, intensive care unit; NA, not applicable.

^a^ Covariables are the same as shown in Table 3.

### Sensitivity Analysis for Mortality Using Different Statistical Approaches

Table 3 summarizes the different statistical approaches used to assess the association of mortality with type of splenic surgery. In the multivariable regression analysis (for covariables refer to Table 3), splenic repair was independently associated with lower mortality compared with patients who underwent splenectomy (aOR, 0.6; 95% CI, 0.4 to 0.9; *P* = .01). PSM (ATE, −0.05; 95% CI, − 0.09 to −0.02; *P* = .003) and IPW (ATE, −0.05; 95% CI, − 0.09 to −0.01; *P* = .009) also showed a lower mortality for patients who underwent splenic repair (Table 3). eTable 4 in Supplement 1 shows the balancing of PSM vs IPW analysis.

## Discussion

The concept of selective nonoperative management in splenic injuries has evolved over recent decades.^11^ As a consequence, operative therapy is performed less frequently and is usually restricted to high-grade injuries. In contrast, there has been a common practice for liberal use of splenectomy in patients with traumatic injury undergoing laparotomy.^5,7^ At present, efforts to preserve an injured spleen during laparotomy after traumatic injury are limited, although publications decades ago documented the feasibility and safety of splenic preservation procedures.^12,13,14,15,16^

The aim of this cohort study was to evaluate the association of surgical strategy (splenic repair vs splenectomy) for traumatic splenic injuries with outcomes. Interestingly, we identified a negative association between splenic repair surgery (vs splenectomy) and in-hospital mortality. Moreover, splenic repair failed in only approximately 1 of 20 patients. In these patients, the mortality rate was similar to that for patients who underwent initial splenectomy.

Comparing outcomes in patients who underwent splenic repair vs splenectomy for trauma laparotomy is difficult: the relatively small number of patients included in previous retrospective studies and the inhomogeneity of injury characteristics and severity between the 2 groups complicate the assessment of outcomes between the 2 groups. More than 40 years ago, Traub et al^17^ evaluated 272 patients for splenic trauma at a single institution. Overall, 41 patients underwent splenic preservation. One of those patients required the return to the operating room, because of a missed hilar laceration at the original laparotomy. Mortality (23.4% vs 4.9%) and sepsis (8.7% vs 4.9%) were more common in the splenectomy group. However, patients with splenectomy also sustained more chest, spine, intraabdominal, and vascular injuries, accounting for the higher mortality.^17^

A recent study^18^ compared patients with splenorrhaphy with those who underwent splenectomy. The authors excluded patients with a GCS score less than 9 to prevent substantial confounding. In the multivariable logistic regression analysis, patients who underwent splenectomy had an aOR of 2.3 (95% CI 7.82-2.92) for mortality compared with those who underwent splenorrhapy.^18^ However, the multivariable regression analysis only corrected for a limited number of potential confounders. In particular, associated injuries were not sufficiently adjusted, because only a total injury AIS score was considered, without defining this score in detail. A single score hardly accounted for the significant differences in injury severity between the 2 groups, with an Injury Severity Score of 26 in the splenectomy group and 17.8 in the splenorrhaphy group.^18^ Finally, the study did not consider the timing of the splenic procedure: immediate splenectomy was categorized the same as a splenectomy performed 5 days after admission, although the latter procedure should correctly be classified as a failed nonoperative procedure.^18^

The spleen is a complex organ and hosts a wide range of immunological functions alongside its role in hematopoiesis and red blood cell clearance.^19^ Potential benefits of splenic preservation include the maintenance of splenic function while avoiding an increased risk of infection.^20^ Patient education, vaccination, and empirical antibiotic therapy in case of fever are important measures that need to be addressed in the management of patients after splenectomy.^21^ In addition to late infections, Traub et al^17^ reported more than 40 years ago that patients who underwent splenectomy were twice as likely to have sepsis as those who had a splenic preservation. Consistent with those results, recent studies^8,22,23^ have also identified an increased vulnerability to early infections after splenectomy. In line with these findings, our conditional regression analysis after 1:1 matching identified a higher, but not statistically significant, risk of severe sepsis and a significantly lower risk of ICU admission, when splenic repair was performed. Furthermore, there is evidence that splenectomy is associated with increased risk of thromboembolic complications,^9,24^ although our study could not confirm these findings. Frequently encountered systemic reactions, such as early leukocytosis and thrombocytosis, after splenectomy may potentially play a role and affect different outcomes. However, the interactions and consequences of these changes at the cellular level are poorly understood. Further studies are needed, particularly to understand the underlying mechanisms with the potential consequences on outcomes.

It is often argued that the potential advantage of splenectomy over splenic repair is the shorter operative time and lower risk of rebleeding. However, the prolonged surgical times for splenic repair compared with splenectomy could not be confirmed in previous studies.^17^ In addition, other studies^12,13,14,15,16^ have confirmed the safety of splenic repair. Our results confirmed these findings and showed that splenectomy was required in 5.3% of patients after initial splenic repair, and, importantly, even in the case of failed splenic repair, the mortality rate was similar to that of patients who underwent an initial splenectomy.

The selection of type of operative management of the spleen in patients undergoing laparotomy after traumatic injury depends on several factors, including the severity of the injury, the patient’s hemodynamic condition, and the surgeon’s expertise. Factors such as the patient’s age, comorbidities, and the presence of associated injuries may also play a role in the decision-making process. The increasing rate of nonoperative management in splenic injuries may be an important reason why many trauma surgeons today lack experience with various splenic repair techniques. Current guidelines remain very vague on the question when a splenic salvage procedure or a splenectomy should be performed: the World Society for Emergency Surgery considers both procedures, splenectomy and splenic salvage, as options in the event of hemodynamic instability or ineffective angioembolization in splenic trauma.^1^ However, a small retrospective study^25^ conducted almost 10 years ago confirmed the feasibility of an established splenic salvage procedure protocol. Another study by Akinkuolie et al^26^ suggested that major determinants of splenectomy were the grade of the splenic injury and the experience of the surgeon and the assistants. The more experienced the team, the higher the rate of splenic preservation surgery. In addition, splenectomy was more common if the operation was performed at night.^26^ However, it is important to clearly emphasize that in splenic trauma with severe hemorrhagic shock or multiple bleeding organs, including coagulopathy, splenectomy remains the standard-of-care procedure. Patients with stabilized vital signs and repairable splenic injuries should be considered for splenic preservation. Future studies should focus on intraoperative criteria to safely identify patients who can be treated with splenic repair. Technical aspects of splenic preservation should be taught to the younger generation of surgeons.

### Limitations

Several limitations need to be addressed. First this is a retrospective study based on a large database and may face limitations such as data inaccuracies, incomplete records, and reporting biases. The generalizability of the findings should be restricted owing to the specific population and practices captured in the TQIP database, which may not fully represent all trauma care settings.

In addition, the TQIP database does not provide long-term data, so that the occurrence of OPSI in particular could not be accurately recorded. Furthermore, the benefit of splenic preservation compared with splenectomy may be even more apparent in children but was not evaluated in the present study. In addition, patient numbers were too small to perform subanalyses between the different surgical repair procedures (eg, sutures, partial splenectomy, or plastic operations) or restriction to isolated severe splenic injuries. Laparoscopic procedures were not considered for the present study because laparoscopy has been described only in some cases of hemodynamically stable, low-to-moderate grade splenic injuries.^27^ The role of angioembolization in splenic repair procedures was not the focus of the present study and should be evaluated in future projects.

Although an extended matching including the extent of injury between patients with splenectomy vs splenic repair was performed, this remains a crude measure to align the 2 groups and determine which procedure was performed. Although multiple statistical methods were used to adjust for confounding, other factors, such as specific intraoperative findings or the surgeon’s experience, may have been decisive in determining which surgical procedure was performed. This information is not available in the database, nor is specific information on the reason why a procedure was performed (splenectomy vs splenic repair); thus, residual confounding cannot be excluded. In addition, the TQIP database does not provide information about the cause of death, and it is, therefore, difficult to identify the underlying mechanisms leading to the found association with mortality.

## Conclusions

The present study identified a negative association between mortality and splenic repair vs splenectomy in patients who underwent laparotomy for severe traumatic splenic injury. Splenectomy was required for approximately 5% of patients after initial splenic repair. The findings suggest that efforts to preserve the spleen might be indicated in selected cases of severe splenic injuries.

## Notes

Supplement 1. **eAppendix.** Detailed information on extracted variables
eReferences
**eTable 1.** ICD-9 and 10 procedures considered for splenectomy and splenic repair **eTable 2.** Patients’ characteristics, clinical data of patients with severe splenic injury undergoing splenic repair or splenectomy within 6 hours of admission **eTable 3.** Trauma type and injury severity after 1:1 exact matching of patients with severe splenic injury undergoing splenic repair or splenectomy within 6 hours of admission **eTable 4.** Balance over treatment groups (Control = splenectomy; n=10,820 vs. Treated = splenic repair; n=427) after estimation by A) a teffects inverse-probability-weighted (IPW) estimator and B) a teffects propensity-score matching (PSM) estimator

Supplement 2.
Data Sharing Statement
