# Supplementary material for: Surgical Repair vs Splenectomy in Patients With Severe Traumatic Spleen Injuries
Source: JAMA Netw Open. 2024 Aug 2;7(8):e2425300. doi: 10.1001/jamanetworkopen.2024.25300 (PMC11297384; doi:10.1001/jamanetworkopen.2024.25300)
Supplement: Supplement 1. — eAppendix. Detailed information on extracted variables eReferences eTable 1. ICD-9 and 10 procedures considered for splenectomy and splenic repair eTable 2. Patients’ characteristics, clinical data of patients with severe splenic injury undergoing splenic repair or splenectomy within 6 hours of admission eTable 3. Trauma type and injury severity after 1:1 exact matching of patients with severe splenic injury undergoing splenic repair or splenectomy within 6 hours of admission eTable 4. Balance over treatment groups (Control = splenectomy; n=10,820 vs. Treated = splenic repair; n=427) after estimation by A) a teffects inverse-probability-weighted (IPW) estimator and B) a teffects propensity-score matching (PSM) estimator [file jamanetwopen-e2425300-s001.pdf]

Supplemental Online Content

Jakob DA, Müller M, Kolitsas A, Exadaktylos AK, Demetriades D. Surgical repair vs splenectomy in patients with severe traumatic spleen injuries. *JAMA Netw Open*. 2024;7(8):e2425300. doi:10.1001/jamanetworkopen.2024.25300

eAppendix. Detailed information on extracted variables

eReferences

eTable 1. ICD-9 and 10 procedures considered for splenectomy and splenic repair

eTable 2. Patients' characteristics, clinical data of patients with severe splenic injury undergoing splenic repair or splenectomy within 6 hours of admission

eTable 3. Trauma type and injury severity after 1:1 exact matching of patients with severe splenic injury undergoing splenic repair or splenectomy within 6 hours of admission

eTable 4. Balance over treatment groups (Control = splenectomy; n=10,820 vs. Treated = splenic repair; n=427) after estimation by A) a teffects inverse-probability-weighted (IPW) estimator and B) a teffects propensity-score matching (PSM) estimator

This supplemental material has been provided by the authors to give readers additional information about their work.

© 2024 Jakob DA et al. *JAMA Network Open.*

eAppendix. Detailed information on extracted variables

For all patients with traumatic severe splenic injuries undergoing laparotomy the following variables were extracted from the TQIP database:

i) demographic data such as age, gender, race, height (cm) and weight (kg),

ii) comorbidities [steroid use, current smoker, diabetes mellitus, hypertension, history of cerebrovascular accident, respiratory disease, congestive heart failure, myocardial infarction, liver cirrhosis, chronic kidney disease, peripheral arterial disease, disseminated cancer/ on chemotherapy, dementia, substance abuse disorder, bleeding disorder, anticoagulation therapy], admission data [HR, SBP, GCS], mechanism of injury (blunt vs. penetrating) including treatment at a level 1 center,

iii) data on anatomic location and severity of the injury by calculating the AIS for each body region and each patient,

iv) surgical splenic procedures including procedure timing. Procedures were identified from the TQIP database by extracting all ICD 9 and ICD 10 procedure codes associated with open splenic repair and/ or open splenectomy. The ICD 9 and 10 codes used to identify patients with an open splenic repair and/ or a splenectomy procedure are provided in **eTable 1**.

v) data on primary outcome included in hospital mortality; secondary outcomes were complications, intensive care unit (ICU) admission, hospital and total ICU length of stay. The following complications were recorded and summarized as overall complications: thromboembolic events including deep vein thrombosis (DVT) and/ or pulmonary embolism (PE), stroke (cerebrovascular accident), myocardial infarction, acute kidney injury, acute respiratory distress syndrome (ARDS), severe sepsis and unplanned return to operating room.

© 2024 Jakob DA et al. *JAMA Network Open.*

The definitions for all collected variables can be found in the National Trauma Data Standard provided by the ACS[1]. Hypotension was defined as a blood pressure <90 mmHg, and tachycardia was defined as a heart rate >120 beats per minute. Body Mass Index (BMI) was calculated by the patient's documented weight in kilograms divided by the square of height in meters. Obesity was defined by a BMI above 30 kg/m^2 according to the World Health Organization recommendations[2]. Race in the TQIP database is self-reported by patients or identified by a family member and is provided in accordance with the US Census Bureau[3].

eReferences:

1. American College of Surgeons. National Tauma Data Sandard (NTDS). https://www.facs.org/quality-programs/trauma/quality/national-trauma-data-bank/national-trauma-data-standard/. Accessed July 04, 2023.
2. World Health Organization (WHO). Obesity. https://www.who.int/health-topics/obesity. Accessed July 04, 2023.
3. Mukherjee D, Lidor AO, Chu KM, Gearhart SL, Haut ER, Chang DC. Postoperative venous thromboembolism rates vary significantly after different types of major abdominal operations. Journal of gastrointestinal surgery : official journal of the Society for Surgery of the Alimentary Tract. Nov 2008;12(11):2015-22. doi:10.1007/s11605-008-0600-1

© 2024 Jakob DA et al. *JAMA Network Open.*

eTable 1. ICD-9 and 10 procedures considered for splenectomy and splenic repair

<u>Open splenic procedure codes ICD 9</u>
<u>Splenectomy:</u>
41.50 Total splenectomy

<u>Spenic repair:</u>
41.42 Excision of lesion or tissue of spleen
41.43 Partial splenectomy
41.95 Repair and plastic operations on spleen
41.99 Other operations on spleen

<u>Open splenic procedure codes ICD 10</u>
<u>Splenectomy:</u>

07BP0ZZ	Excision of Spleen, Open Approach
07TP0ZZ	Resection of Spleen, Open Approach
1OB89LA	Excision total, spleen using open [abdominal] approach

<u>Splenic repair:</u>

07QP0ZZ	Repair Spleen, Open Approach
1OB13LA	Control of bleeding, spleen open [abdominal] approach using apposition technique [e.g. suturing or NOS]
1OB13LA	Control of bleeding, spleen open [abdominal] approach using device NEC
1OB13LA	Control of bleeding, spleen open [abdominal] approach using fibrin glue
1OB13LA	Control of bleeding, spleen open [abdominal] approach using chemical cautery [e.g. topical thrombin]
1OB87LA	Excision partial, spleen open [abdominal] approach using apposition technique for closure [e.g. suturing]
1OB13PF	Control of bleeding, spleen open posterior [subcostal] approach using apposition technique [e.g. suturing]

© 2024 Jakob DA et al. *JAMA Network Open.*

eTable 2. Patients' characteristics, clinical data of patients with severe splenic injury undergoing splenic repair or splenectomy within 6 hours of admission

	Total (n=11,247)		Splenectomy (n=10,820)		Splenic repair (n=427)		P-value*
**DEMOGRAPHICS**							
**Age (years)**	35	[24; 52]	35	[24; 52]	30	[23; 44]	<0.001
**Age groups**							
16-45	7,453	[66.3]	7,124	[65.8]	329	[77.0]	<0.001
>45-65	2,817	[25.0]	2,745	[25.4]	72	[16.9]	
>65-75	636	[5.7]	621	[5.7]	15	[3.5]	
>75	341	[3.0]	330	[3.0]	11	[2.6]	
**Sex**							
Male	8,179	[72.7]	7,860	[72.6]	319	[74.7]	0.35
Female	3,068	[27.3]	2960	[27.4]	225	[25.3]	0.35
**Docum. BMI>30kg/m^2**	2,639	[23.5]	2,545	[23.5]	94	[22.0]	0.47
**Race**							
White	7,885	[70.1]	7,644	[70.6]	241	[56.4]	<0.001
Black	1,835	[16.3]	1,734	[16.0]	101	[23.7]	
Asian	174	[1.5]	167	[1.5]	7	[1.6]	
Other‡	1,353	[12.0]	1,275	[11.8]	78	[18.3]	
**Level 1 trauma center treatment**	6,237	[55.6]	6,007	[55.7]	230	[54.0]	0.49
**VITALS**							
**Hypotension [<90mmHg]**	2,671	[23.7]	2,603	[24.1]	68	[15.9]	<0.001
**Tachycardia [HR>120bpm]**	3,289	[29.2]	3,192	[29.5]	97	[22.7]	0.003
**GCS**	15	[8; 15]	15	[8; 15]	15	[14; 15]	<0.001
**COMORBIDITIES**							
**Any comorbidities**	5199	[46.2]	5004	[46.2]	195	[45.7]	0.814
**Steroid use**	34	[0.3]	33	[0.3]	1	[0.2]	0.79
**Current smoker**	2,610	[23.2]	2,514	[23.2]	96	[22.5]	0.72
**Diabetes mellitus**	651	[5.8]	629	[5.8]	22	[5.2]	0.57
**Hypertension**	1,616	[14.4]	1,562	[14.4]	54	[12.6]	0.30
**Cerebrovascular accident**	61	[0.5]	58	[0.5]	3	[0.7]	0.65
**Respiratory disease**	396	[3.5]	382	[3.5]	14	[3.3]	0.78
**Congestive heart failure**	116	[1.0]	114	[1.1]	2	[0.5]	0.24
**Myocardial infarction (past)**	44	[0.4]	43	[0.4]	1	[0.2]	0.60
**Liver cirrhosis**	201	[1.8]	200	[1.8]	1	[0.2]	0.01
**Chronic kidney disease**	38	[0.3]	35	[0.3]	3	[0.7]	0.19
**Peripheral arterial disease**	30	[0.3]	28	[0.3]	2	[0.5]	0.41
**Dissemin. cancer/ on chemotherapy**	34	[0.3]	33	[0.3]	1	[0.2]	0.79
**Dementia**	28	[0.2]	27	[0.2]	1	[0.2]	0.95

**Substance abuse disorder**	1,869	[16.6]	1,797	[16.6]	72	[16.9]	0.89
**Bleeding disorder**	222	[2.0]	214	[2.0]	8	[1.9]	0.88
**Anticoagulant therapy**	142	[1.3]	139	[1.3]	3	[0.7]	0.29
**TRAUMA TYPE**							
**Trauma type***							
Blunt	8,998	[80.0]	8,783	[81.2]	215	[50.4]	<0.001
Penetrating	2,202	[19.6]	1,991	[18.4]	211	[49.4]	
Unspecified/ missing	47	[0.4]	46	[0.4]	1	[0.2]	
**INJURY SEVERITY**							
**AIS head**	0	[0; 2]	0	[0; 2]	0	[0; 1]	<0.001
**AIS face**	0	[0; 1]	0	[0; 1]	0	[0; 0]	<0.001
**AIS neck**	0	[0; 0]	0	[0; 0]	0	[0; 0]	0.16
**AIS thorax**	3	[2; 3]	3	[2; 3]	3	[2; 3]	0.55
**AIS spleen**	4	[3; 5]	4	[3; 5]	3	[3; 4]	<0.001
**AIS abdomen (no spleen)**	2	[0; 3]	2	[0; 3]	2	[0; 3]	0.15
**AIS spine**	0	[0; 2]	0	[0; 2]	0	[0; 2]	0.005
**AIS lower extremity**	1	[0; 2]	1	[0; 2]	0	[0; 2]	<0.001
**AIS upper extremity**	0	[0; 2]	0	[0; 2]	0	[0; 1]	<0.001
**AIS external, other, unspec.**	0	[0; 0]	0	[0; 0]	0	[0; 0]	0.29
**ISS**	34	[25; 42]	34	[25; 42]	26	[18; 34]	<0.001
**Isolated[#] severe splenic injury**	1,775	[15.8]	1,716	[15.8]	62	[14.5]	0.47
**COMPLICATIONS**							
**Any complications**	2,465	[21.9]	2,391	[22.1]	74	[17.3]	0.02
**Thromboembolic event**	757	[6.7]	737	[6.8]	20	[4.7]	0.09
**Deep vein thrombosis**	549	[4.9]	538	[5.0]	11	[2.6]	0.02
**Pulmonary embolism**	280	[2.5]	270	[2.5]	10	[2.3]	0.84
**Stroke**	119	[1.1]	116	[1.1]	3	[0.7]	0.46
**Myocardial infarction**	62	[0.6]	60	[0.6]	2	[0.5]	0.81
**Acute kidney injury**	503	[4.5]	488	[4.5]	15	[3.5]	0.33
**ARDS**	406	[3.6]	397	[3.7]	9	[2.1]	0.09
**Pneumonia**	919	[8.2]	887	[8.2]	32	[7.5]	0.60
**Severe sepsis**	300	[2.7]	290	[2.7]	10	[2.3]	0.67
**Unplanned return to OR**	603	[5.4]	583	[5.4]	20	[4.7]	0.53
**OUTCOME**							
**LOS hospital [days]**	10	[6; 20]	10	[6; 20]	10	[6; 18]	0.56
**ICU treatment needed**	9,644	[85.7]	9,302	[86.0]	342	[80.1]	0.001
**ICU treatment [days]**	4	[2; 11]	4	[2; 11]	4	[1; 9]	0.002
**In-hospital mortality**	1,828	[16.3]	1,795	[16.6]	33	[7.7]	<0.001

Abbreviations: BMI, Body Mass Index; SBP, Systolic Blood Pressure; HR, Heart Rate; GCS, Glascow Coma Scale; AIS, Abbreviated Injury Scale; unspec., unspecified; ISS, Injury Severity Score; ARDS, Acute Respiratory Distress Syndrome; OR, Operating Room; LOS, Length Of Stay; ICU, Intensive Care Unit.

© 2024 Jakob DA et al. *JAMA Network Open.*

‡races not classified as white, black or asian were classified as other race
#all AIS-non-splenic-groups ≤ 2; * p-values obtained with Chi-squared for binary outcomes respectively with Wilcoxon rank sum test for continuous outcomes.

© 2024 Jakob DA et al. *JAMA Network Open.*

eTable 3. Trauma type and injury severity after 1:1 exact matching of patients with severe splenic injury undergoing splenic repair or splenectomy within 6 hours of admission

	Splenectomy (n=400)		Splenic repair (n=400)		P-value*
**TRAUMA TYPE**					
Blunt	197	[49.2]	198	[49.5]	1.00
Penetrating	201	[50.2]	201	[50.2]	1.00
Unspecified/ missing	2	[0.5]	1	[0.2]	1.00
**INJURY SEVERITY**					
**AIS Spleen**					
3	250	[62.5]	250	[62.5]	1.00
4	104	[26.0]	104	[26.0]	
5	46	[11.5]	46	[11.5]	
**AIS head**					
0-2	358	[89.5]	358	[89.5]	1.00
3	15	[3.8]	15	[3.8]	1.00
4-5	27	[6.8]	27	[6.8]	1.00
**AIS face**					
0-2	397	[99.2]	399	[99.8]	0.63
3	2	[0.5]	1	[0.2]	1.00
4-5	1	[0.2]	0	[0.0]	1.00
**AIS neck**					
0-2	399	[99.8]	399	[99.8]	1.00
3	1	[0.2]	1	[0.2]	1.00
**AIS thorax**					
0-2	113	[28.2]	113	[28.2]	1.00
3	210	[52.5]	210	[52.5]	1.00
4-5	77	[19.1]	77	[19.1]	1.00
**AIS abdomen (excl. spleen)**					
0-2	231	[57.8]	245	[61.3]	0.33
3	91	[22.8]	97	[24.2]	0.67
4-5	78	[19.5]	58	[14.5]	0.07
**AIS spine**					
0-2	391	[97.8]	391	[97.8]	1.00
3	5	[1.2]	5	[1.2]	1.00
4-5	4	[1.0]	4	[1.0]	1.00
**AIS lower extremity**					
0-2	332	[83.0]	332	[83.0]	1.00
3	61	[15.2]	61	[15.2]	1.00
4-5	7	[1.8]	7	[1.8]	1.00
**AIS upper extremity**					

© 2024 Jakob DA et al. *JAMA Network Open.*

0-2	393	[98.2]	393	[98.2]	1.00
3	7	[1.8]	7	[1.8]	1.00
**AIS external, other, unspec.**					
0-2	400	[100.0]	400	[100.0]	1.00
**ISS**	25.5	[19; 34]	25	[18; 34]	0.13

Abbreviations: AIS, Abbreviated Injury Scale; unspec., unspecified; ISS, Injury Severity Score

400 out of 427 (93.7%) patients with splenic repair were 1:1 matched with control patients who had splenectomy within 6 hours of admission. The controls were matched on age groups (16-45; >45-65; >65-75; >75 years), sex, hypotension [<90mmHg] on admission, penetrating trauma mechanism as well as AIS spleen grades 3,4,5, AIS groups (AIS 0-2, AIS 3, AIS 4-5) for head, face, neck, thorax, spine, and lower & upper extremity.

* p-values were calculated using Wilcoxon signed rank test and McNemars test (exact p-values).

© 2024 Jakob DA et al. *JAMA Network Open.*

eTable 4. Balance over treatment groups (Control = splenectomy; n=10,820 vs. Treated = splenic repair; n=427) after estimation by A) a teffects inverse-probability-weighted (IPW) estimator and B) a teffects propensity-score matching (PSM) estimator

A) IPW

	Means		Variances		Standardized differences	
**DEMOGRAPHICS**	**Control**	**Treated**	**Control**	**Treated**	**Raw**	**Weighted**
**Age (years)**						
**Age groups**	39.18	35.32	295.81	258.36	0.23	0.11
16-45	0.66	0.77	0.22	0.18	0.25	0.14
>45-65	0.25	0.17	0.19	0.14	0.21	0.16
>65-75	0.06	0.04	0.05	0.03	0.11	0.02
>75	0.03	0.03	0.03	0.03	0.03	0.01
**Sex, male**	0.73	0.75	0.20	0.19	0.05	0.12
**Docum. BMI>30kg/m^2**	0.24	0.22	0.18	0.17	0.04	0.09
**Race**						
White	0.71	0.56	0.21	0.25	0.30	0.03
Black	0.16	0.24	0.13	0.18	0.19	0.09
Asian	0.02	0.02	0.02	0.02	0.01	0.01
Other	0.12	0.18	0.10	0.15	0.18	0.04
**VITALS**						
**Hypotension [<90mmHg]**	0.24	0.16	0.18	0.13	0.20	0.08
**Tachycardia [HR>120bpm]**	0.30	0.23	0.21	0.18	0.15	0.04
**GCS**	11.62	12.98	23.71	15.81	0.31	0.09
**COMORBIDITIES**						
**Any comorbidities**	0.46	0.46	0.25	0.25	0.01	0.11
**Steroid use**	0.00	0.00	0.00	0.00	0.01	0.02
**Current smoker**	0.23	0.22	0.18	0.17	0.02	0.17
**Diabetes mellitus**	0.06	0.05	0.05	0.05	0.03	0.13
**Hypertension**	0.14	0.13	0.12	0.11	0.05	0.01
**Cerebrovascular accident**	0.01	0.01	0.01	0.01	0.02	0.06
**Respiratory disease**	0.04	0.03	0.03	0.03	0.01	0.05
**Congestive heart failure**	0.01	0.00	0.01	0.00	0.07	0.09
**Myocardial infarction (past)**	0.00	0.00	0.00	0.00	0.03	0.02
**Liver cirrhosis**	0.02	0.00	0.02	0.00	0.16	0.05
**Chronic kidney disease**	0.00	0.01	0.00	0.01	0.05	0.01
**Peripheral arterial disease**	0.00	0.00	0.00	0.00	0.03	0.00
**Dissemin. cancer/on chemotherapy**	0.00	0.00	0.00	0.00	0.01	0.10
**Dementia**	0.00	0.00	0.00	0.00	0.00	0.03
**Substance abuse disorder**	0.17	0.17	0.14	0.14	0.01	0.09
**Bleeding disorder**	0.02	0.02	0.02	0.02	0.01	0.03
**Anticoagulant therapy**	0.01	0.01	0.01	0.01	0.06	0.11
**TRAUMA TYPE**						
Blunt	0.81	0.50	0.15	0.25	0.69	0.06

© 2024 Jakob DA et al. *JAMA Network Open.*

Penetrating	0.18	0.49	0.15	0.25	0.69	0.05
Unspecified/ missing	0.00	0.00	0.00	0.00	0.03	0.07
**INJURY SEVERITY**						
**AIS head**	1.21	0.73	2.79	1.85	0.31	0.02
**AIS face**	0.45	0.30	0.53	0.38	0.21	0.01
**AIS neck**	0.10	0.07	0.25	0.15	0.08	0.08
**AIS thorax**	0.83	0.69	1.38	1.33	0.12	0.09
**AIS spleen**	4.06	3.48	0.63	0.47	0.79	0.06
**AIS abdomen (no spleen)**	1.92	2.02	2.47	2.17	0.07	0.07
**AIS spine**	2.59	2.64	2.00	1.76	0.03	0.13
**AIS lower extremity**	1.22	0.99	1.89	1.77	0.17	0.03
**AIS upper extremity**	0.85	0.64	0.97	0.82	0.22	0.02
**AIS external, other, unspec.**	0.11	0.09	0.10	0.08	0.06	0.02
**ISS**	34.01	27.73	164.72	149.92	0.50	0.04
**COMPLICATIONS**						
**Any complications**	0.22	0.17	0.17	0.14	0.12	0.00
**Thromboembolic event**	0.07	0.05	0.06	0.04	0.09	0.00
**Deep vein thrombosis**	0.05	0.03	0.05	0.03	0.13	0.00
**Pulmonary embolism**	0.02	0.02	0.02	0.02	0.01	0.00
**Stroke**	0.01	0.01	0.01	0.01	0.04	0.04
**Myocardial infarction**	0.01	0.00	0.01	0.00	0.01	0.01
**Acute kidney injury**	0.05	0.04	0.04	0.03	0.05	0.05
**ARDS**	0.04	0.02	0.04	0.02	0.09	0.10
**Pneumonia**	0.08	0.07	0.08	0.07	0.03	0.06
**Severe sepsis**	0.03	0.02	0.03	0.02	0.02	0.01
**Unplanned return to OR**	0.05	0.05	0.05	0.04	0.03	0.05
**OUTCOME**						
**LOS hospital [days]**	15.49	14.93	260.15	257.66	0.04	0.04
**ICU treatment needed**	0.86	0.80	0.12	0.16	0.16	0.05
**ICU treatment [days]**	8.24	7.32	108.48	137.78	0.08	0.03
**In-hospital mortality**	0.17	0.08	0.14	0.07	0.27	0.16

B) PSM

	**Means**		**Variances**		**Standardized differences**	
**DEMOGRAPHICS**	**Control**	**Treated**	**Control**	**Treated**	**Raw**	**Matched**
**Age (years)**	39.18	35.32	295.81	258.36	0.23	0.13
**Age groups**						
16-45	0.66	0.77	0.22	0.18	0.25	0.19
>45-65	0.25	0.17	0.19	0.14	0.21	0.25
>65-75	0.06	0.04	0.05	0.03	0.11	0.10
>75	0.03	0.03	0.03	0.03	0.03	0.00
**Sex, male**	0.73	0.75	0.20	0.19	0.05	0.07
**Docum. BMI>30kg/m^2**	0.24	0.22	0.18	0.17	0.04	0.05
**Race**						
White	0.71	0.56	0.21	0.25	0.30	0.03
Black	0.16	0.24	0.13	0.18	0.19	0.05
Asian	0.02	0.02	0.02	0.02	0.01	0.05

© 2024 Jakob DA et al. *JAMA Network Open.*

Other	0.12	0.18	0.10	0.15	0.18	0.08
**VITALS**						
**Hypotension [<90mmHg]**	0.24	0.16	0.18	0.13	0.20	0.06
**Tachycardia [HR>120bpm]**	0.30	0.23	0.21	0.18	0.15	0.05
**GCS**	11.62	12.98	23.71	15.81	0.31	0.06
**COMORBIDITIES**						
**Any comorbidities**	0.46	0.46	0.25	0.25	0.01	0.14
**Steroid use**	0.00	0.00	0.00	0.00	0.01	0.01
**Current smoker**	0.23	0.22	0.18	0.17	0.02	0.19
**Diabetes mellitus**	0.06	0.05	0.05	0.05	0.03	0.12
**Hypertension**	0.14	0.13	0.12	0.11	0.05	0.01
**Cerebrovascular accident**	0.01	0.01	0.01	0.01	0.02	0.04
**Respiratory disease**	0.04	0.03	0.03	0.03	0.01	0.07
**Congestive heart failure**	0.01	0.00	0.01	0.00	0.07	0.08
**Myocardial infarction (past)**	0.00	0.00	0.00	0.00	0.03	0.01
**Liver cirrhosis**	0.02	0.00	0.02	0.00	0.16	0.06
**Chronic kidney disease**	0.00	0.01	0.00	0.01	0.05	0.03
**Peripheral arterial disease**	0.00	0.00	0.00	0.00	0.03	0.04
**Dissemin. cancer/on chemotherapy**	0.00	0.00	0.00	0.00	0.01	0.10
**Dementia**	0.00	0.00	0.00	0.00	0.00	0.02
**Substance abuse disorder**	0.17	0.17	0.14	0.14	0.01	0.09
**Bleeding disorder**	0.02	0.02	0.02	0.02	0.01	0.01
**Anticoagulant therapy**	0.01	0.01	0.01	0.01	0.06	0.13
**TRAUMA TYPE**						
Blunt	0.81	0.50	0.15	0.25	0.69	0.02
Penetrating	0.18	0.49	0.15	0.25	0.69	0.03
Unspecified/ missing	0.00	0.00	0.00	0.00	0.03	0.07
**INJURY SEVERITY**						
**AIS head**	1.21	0.73	2.79	1.85	0.31	0.05
**AIS face**	0.45	0.30	0.53	0.38	0.21	0.00
**AIS neck**	0.10	0.07	0.25	0.15	0.08	0.09
**AIS thorax**	0.83	0.69	1.38	1.33	0.12	0.07
**AIS spleen**	4.06	3.48	0.63	0.47	0.79	0.02
**AIS abdomen (no spleen)**	1.92	2.02	2.47	2.17	0.07	0.06
**AIS spine**	2.59	2.64	2.00	1.76	0.03	0.23
**AIS lower extremity**	1.22	0.99	1.89	1.77	0.17	0.04
**AIS upper extremity**	0.85	0.64	0.97	0.82	0.22	0.11
**AIS external, other, unspec.**	0.11	0.09	0.10	0.08	0.06	0.01
**ISS**	34.01	27.73	164.72	149.92	0.50	0.05
**COMPLICATIONS**						
**Any complications**	0.22	0.17	0.17	0.14	0.12	0.05
**Thromboembolic event**	0.07	0.05	0.06	0.04	0.09	0.04
**Deep vein thrombosis**	0.05	0.03	0.05	0.03	0.13	0.02
**Pulmonary embolism**	0.02	0.02	0.02	0.02	0.01	0.05
**Stroke**	0.01	0.01	0.01	0.01	0.04	0.05
**Myocardial infarction**	0.01	0.00	0.01	0.00	0.01	0.02
**Acute kidney injury**	0.05	0.04	0.04	0.03	0.05	0.00

© 2024 Jakob DA et al. *JAMA Network Open.*

**ARDS**	0.04	0.02	0.04	0.02	0.09	0.06
**Pneumonia**	0.08	0.07	0.08	0.07	0.03	0.05
**Severe sepsis**	0.03	0.02	0.03	0.02	0.02	0.01
**Unplanned return to OR**	0.05	0.05	0.05	0.04	0.03	0.03
**OUTCOME**						
**LOS hospital [days]**	15.49	14.93	260.15	257.66	0.04	0.12
**ICU treatment needed**	0.86	0.80	0.12	0.16	0.16	0.04
**ICU treatment [days]**	8.24	7.32	108.48	137.78	0.08	0.07
**In-hospital mortality**	0.17	0.08	0.14	0.07	0.27	0.20

Although there is no clear consensus, some researchers suggest that a standardized difference of <0.1 suggest no relevant imbalance in the baseline covariate (Austin 2009, doi: 10.1002/sim.3697).

© 2024 Jakob DA et al. *JAMA Network Open.*
