# Supplementary material for: Surgical Repair vs Splenectomy in Patients With Severe Traumatic Spleen Injuries
Source: JAMA Netw Open. 2024 Aug 2;7(8):e2425300. doi: 10.1001/jamanetworkopen.2024.25300 (PMC11297384; doi:10.1001/jamanetworkopen.2024.25300)
Supplement: Supplement 2. — Data Sharing Statement [file jamanetwopen-e2425300-s002.pdf]

Data Sharing Statement

Jakob. Surgical Repair vs Splenectomy in Patients With Severe Traumatic Spleen Injuries. *JAMA Netw Open*. Published August 02, 2024. doi:10.1001/jamanetworkopen.2024.25300

Data
Data available: No

Additional Information
Explanation for why data not available: Data available: no Additional Information Explanation for why data not available: The data used for this study are provided by the American College of Surgeons and may be obtained with a license at https://www.facs.org/quality-programs/trauma/quality/trauma-quality-improvement-program
